# Glycosylation site prediction using ensembles of Support Vector Machine classifiers

**DOI:** 10.1186/1471-2105-8-438

**Published:** 2007-11-09

**Authors:** Cornelia Caragea, Jivko Sinapov, Adrian Silvescu, Drena Dobbs, Vasant Honavar

**Affiliations:** 1 Artificial Intelligence Research Laboratory, Computer Science Department, Iowa State University, USA; 2 Center for Computational Intelligence, Learning, and Discovery, Iowa State University, USA; 3 Department of Genetics, Development and Cell Biology, Iowa State University, USA; 4 Bioinformatics and Computational Biology Program, Iowa State University, USA

## Abstract

**Background:**

Glycosylation is one of the most complex post-translational modifications (PTMs) of proteins in eukaryotic cells. Glycosylation plays an important role in biological processes ranging from protein folding and subcellular localization, to ligand recognition and cell-cell interactions. Experimental identification of glycosylation sites is expensive and laborious. Hence, there is significant interest in the development of computational methods for reliable prediction of glycosylation sites from amino acid sequences.

**Results:**

We explore machine learning methods for training classifiers to predict the amino acid residues that are likely to be glycosylated using information derived from the target amino acid residue and its sequence neighbors. We compare the performance of Support Vector Machine classifiers and ensembles of Support Vector Machine classifiers trained on a dataset of experimentally determined N-linked, O-linked, and C-linked glycosylation sites extracted from O-GlycBase version 6.00, a database of 242 proteins from several different species. The results of our experiments show that the ensembles of Support Vector Machine classifiers outperform single Support Vector Machine classifiers on the problem of predicting glycosylation sites in terms of a range of standard measures for comparing the performance of classifiers. The resulting methods have been implemented in *EnsembleGly*, a web server for glycosylation site prediction.

**Conclusion:**

*Ensembles of Support Vector Machine classifiers *offer an accurate and reliable approach to automated identification of putative glycosylation sites in glycoprotein sequences.

## Background

Glycosylation is one of the most complex and ubiquitous post-translational modifications (PTMs) of proteins in eukaryotic cells. It is a dynamic enzymatic process in which saccharides are attached to proteins or lipoproteins, usually on serine (S), threonine (T), asparagine (N), and tryptophan (W) residues. Glycosylation, like phosphorylation, is clinically important because of its role in a wide variety of cellular, developmental and immunological processes, including protein folding, protein trafficking and localization, cell-cell interactions, and epitope recognition [1-8].

Glycosylation can be classified into four types based on the nature of chemical linkage between specific acceptor residues in the protein and sugar: N-linked and O-linked glycosylation, C-mannosylation, and GPI (glycosylphosphatidylinositol) anchors. The acceptor residues represent the glycosylation sites.

In *N-linked glycosylation*, the oligosaccharide chain (a.k.a. glycan) is attached to the amide nitrogen of asparagine (Asp, N), which is part of characteristic sequence motifs *N-X-T *(very often), *N-X-S *(often) or *N-X-C *(very rare), where *X *can be any residue except proline [9]. These sequence motifs are necessary, but not sufficient for an Asp residue to serve as an acceptor site for glycan attachment. A variety of different glycans (e.g., N-acetylglucosamine, N-acetylgalactosamine, fucose) can be attached to Asp.

In *O-linked glycosylation*, the glycan is attached to the hydroxyl oxygen of serine (Ser, S) or threonine (Thr, T). No specific sequence motifs have been defined for O-linked glycosylation. However, it has been reported that most O-linked glycosylation occurs on Ser or Thr residues in close proximity to a proline residue [10,11]. Examples of the O-glycans include: O-N-acetylgalactosamine (O-GalNAc) (a.k.a. mucin type), O-N-acetylglucosamine (O-GlcNAc), O-Fucose, O-Glucose, O-Mannose, O-Hexose, O-Xylose. It is important to note that O-GlcNAc glycans are often added to Ser/Thr residues that would otherwise be phosphorylated, one illustration of the complex interplay among eukaryotic post-translational modification systems.

In *C-mannosylation*, the glycan is attached to the carbon of a tryptophan (Trp, W) residue rather than to the amide nitrogen of Asp, or hydroxyl oxygen of Ser or Thr, making it an unusual modification. The attachment occurs within the sequence motifs *W-X-X-W *on the first Trp (W), *W-X-X-C *or *W-X-X-F *[12,13]. We will refer to this type of glycosylation as C-linked glycosylation.

In *GPI anchors *(glycosylphosphatidylinositol or "lipid" anchor), a hydrophobic phosphatidylinositol group is linked to a residue at or near the C-terminus of a protein through a carbohydrate-containing linker. GPI anchor addition is both structurally and functionally related to another important post-translational modification, *prenylation*, in which hydrophobic farnesyl or geranyl-geranyl moieties are added to C-terminal cysteine (Cys, C) residues of target proteins. GPI anchors target and "anchor" proteins to the cell membrane [14].

Experimental determination of glycosylation sites in proteins is an expensive and laborious process [15]. Hence, there is significant interest in computational approaches to reliably predicting the glycosylation sites from an amino acid sequence. Machine learning methods currently offer one of the most cost-effective approaches to construction of predictive models in applications where representative training data are available. Fortunately, O-GlycBase [16] provides such a dataset for training classifiers for predicting glycosylation sites.

From a machine learning point of view, the problem of glycosylation site prediction can be formulated as a binary classification problem: Given a protein sequence *S *of length *N*, *S *= *s*_1 _*s*_2 _⋯ *s*_*N *_over the alphabet Σ of amino acids, |*Σ*| = 20, *s*_*i *_∈ Σ, *i *= 1, ⋯, *N *and *S *∈ Σ*, the task is to predict whether or not a site is a glycosylation site. Machine learning algorithms can then be used to train such classifiers. We train *Support Vector Machines *and *ensembles of Support Vector Machine classifiers *[17,18] to predict glycosylation versus non-glycosylation sites for N-, O-, and C-linked glycosylation types. O-GlycBase dataset does not contain information about GPI anchors.

Several approaches to predicting glycosylation sites have been reported in the literature. Blom et al. [19] provide a review of available prediction methods, databases and servers for glycosylation. Elhammer et al. [20] use information derived from the frequency of amino acids in the neighborhood of a glycosylation site to identify putative glycosylation sites. This method uses only information derived from the sequence neighbors of glycosylated sites, while ignoring the information available from non-glycosylated sites, which might be useful in extracting sequence features that help distinguish glycosylation sites from non-glycosylation sites. Hansen et al. [21] use Artificial Neural Networks trained on information derived from both glycosylation and non-glycosylation sites. Their server, *netOglyc*, makes predictions for mucin type O-linked glycosylation on mammalian proteins. Li et al. [22] train Support Vector Machine classifiers based on physicochemical properties of amino acids and a 0/1 system to classify mucin type O-linked glycosylation on mammalian proteins.

Although work on predicting glycosylation sites exists in the literature, there is significant room for improvement of current approaches.

One particular challenge in training classifiers using standard machine learning algorithms comes from the fact that the available dataset is highly *unbalanced *[23]: the fraction of glycosylation sites is relatively small compared to the fraction of non-glycosylation sites. Classifiers that are trained to optimize accuracy generally perform rather poorly on the minority class. Hence, if accurate classification of sites from the minority class is important (or equivalently, the false positives and false negatives have unequal costs or risks associated with them), a common approach is to change the distribution of glycosylation and non-glycosylation sites during training by randomly selecting a subset of the training data for the majority class. However, this makes it difficult to reliably identify the majority of the glycosylation sites without falsely predicting non-glycosylation sites as glycosylation sites. In addition, this approach fails to utilize all of the information available in the training data extracted from the original sequence dataset.

Against this background, we explore an *ensemble of Support Vector Machine classifiers *[17,18], trained on the "natural" distribution of the data extracted from the original sequence data, for predicting glycosylation sites and we compare it with single Support Vector Machine classifiers.

## Results

The main result of our study is that the ensembles of Support Vector Machine classifiers described here outperform single Support Vector Machine classifiers on the problem of predicting glycosylation sites.

### An ensemble of Support Vector Machines outperforms a single Support Vector Machine trained on unbalanced data on the glycosylation site prediction task

For each glycosylation type considered in this study, N-, O-, and C-linked glycosylation, we trained ensembles of Support Vector Machine (SVM) classifiers to predict whether or not a site in a protein sequence is a glycosylation site. An ensemble of SVMs [17,18] is simply a collection of SVM classifiers, each trained on a *balanced *subsample of the training data. The prediction of the ensemble of SVMs is computed from the predictions of the individual SVM classifiers (see Methods section for further details).

We compared the performance of the ensemble of SVM classifiers with that of a single SVM classifier trained on the original distribution of the glycosylation data (unbalanced data). Note that the ensemble of SVMs is trained on the original distribution of the glycosylation data. With any classifier, it is possible to tradeoff the rate of *true positive *predictions (sensitivity) against the rate of false positive predictions. Hence, it is much more informative to compare the Receiver Operating Characteristic (ROC) curves which show the tradeoff between true positive and false positive predictions over their entire range of possible values than to compare the performance of the classifiers for a particular choice of the tradeoff (which corresponds to a specific point *θ *on the ROC curve) [24].

**Figure 1:**
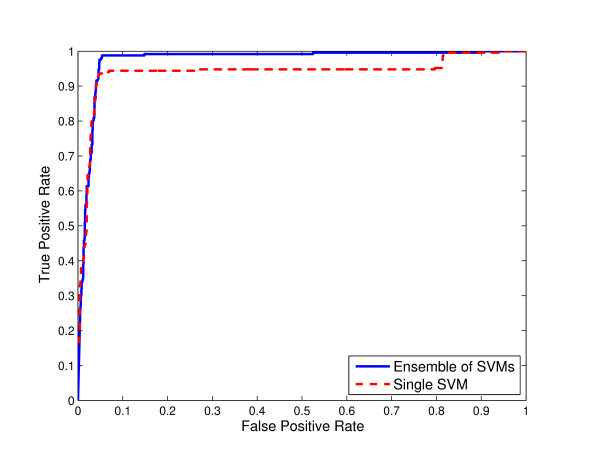
**Comparison of ensemble of SVMs and single SVM from unbalanced data for N-linked glycosylation using local sequence identity**. ROC curves for ensemble of SVMs and single SVM trained on the "natural" distribution of the data extracted from the original glycoprotein sequence dataset for N-linked glycosylation using local sequence identity with 0/1 String Kernel.

**Figure 2:**
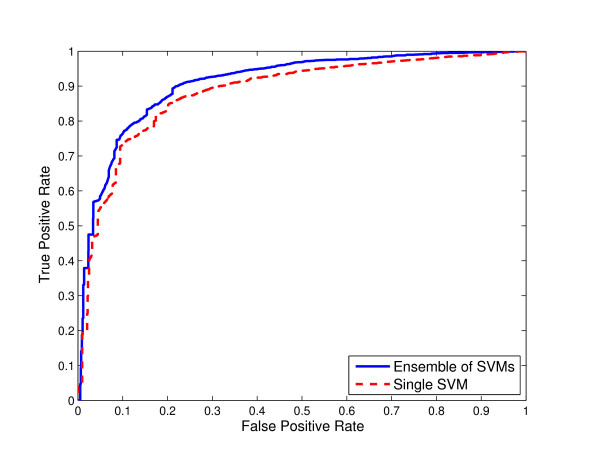
**Comparison of ensemble of SVMs and single SVM from unbalanced data for O-linked glycosylation using local sequence identity**. ROC curves for ensemble of SVMs and single SVM trained on the "natural" distribution of the data extracted from the original glycoprotein sequence dataset for O-linked glycosylation using local sequence identity with 0/1 String Kernel.

**Figure 3:**
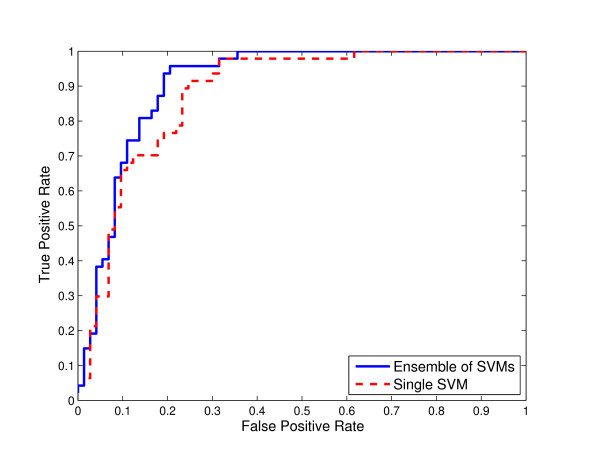
**Comparison of ensemble of SVMs and single SVM from unbalanced data for C-linked glycosylation using local sequence identity**. ROC curves for ensemble of SVMs and single SVM trained on the "natural" distribution of the data extracted from the original glycoprotein sequence dataset for C-linked glycosylation using local sequence identity with 0/1 String Kernel.

Thus, we compared the ROC curves for both ensemble of SVMs and single SVM trained on unbalanced data using local sequence information (the amino acid identity) with 0/1 String Kernel, for N-, O-, and C-linked glycosylation prediction tasks. The ROC curves of ensembles of SVM classifiers for N-linked, O-linked, and C-linked glycosylation sites *dominate *the ROC curves for their single SVM counterparts (Figures 1, 2, and 3 respectively). That is, for any choice of false positive rate, the ensemble of SVMs offers a higher *true positive rate *than the single SVM for the same task.

For N-, O-, and C-linked glycosylation prediction tasks, the Area Under the ROC Curve (AUC) [25] is larger for the ensemble of SVMs than for the corresponding single SVM (Note that the best classifier has an AUC of 1).

The estimated numbers of true positives (TP), false negatives (FN), false positives (FP), and true negatives (TN) depend on how the classification threshold *θ *on the ROC curve is selected (see Methods section for further details). The information obtained from these numbers can be summarized by several commonly used performance measures (e.g., accuracy, sensitivity, specificity, AUC, etc.) that seek to evaluate the quality of the predictions [24].

In the case of classifiers trained to predict N-linked glycosylation sites which occur in relatively "conserved" motifs, at a false positive rate of 0.1, the corresponding true positive rate of the single SVM is 0.94 whereas that of the ensemble of SVMs is 0.99, i.e., 5% greater sensitivity (Figure 1). For a specific point *θ *= 0.5 on the ROC curves, the estimated numbers TP, FN, FP, and TN of the single SVM are 210, 41, 53, 1377 respectively, whereas those of the ensemble of SVMs are 245, 6, 72, 1358. Hence, the single SVM achieves 0.94 accuracy, 0.78 Matthews correlation coefficient, 0.84 sensitivity, 0.80 specificity, 0.82 F-Measure, and 0.94 AUC, and the ensemble of SVMs achieves 0.95 accuracy, 0.84 Matthews correlation coefficient, 0.98 sensitivity, 0.77 specificity, 0.86 F-Measure, and 0.98 AUC (Table 1).

**Table 1:** Performance of classifiers trained to predict N-linked glycosylation sites

Performance Measure	SingleSVM	EnsembleSVM	BalancedSVM
Accuracy	0.94	**0.95**	0.94
MCC	0.78	**0.84**	0.77
Sensitivity	0.84	**0.98**	0.82
Specificity	**0.80**	0.77	0.79
F-Measure	0.82	**0.86**	0.81
AUC	0.94	**0.98**	0.97

Results obtained for N-linked glycosylation using single SVM from unbalanced data (singleSVM), ensemble of SVMs (EnsembleSVM), and single SVM from balanced data (BalancedSVM) for the classification threshold *θ *= 0.5 on the output probability of the classifier. The classifiers are trained on information derived from the target amino acid residue and its sequence neighbors.

In the case of classifiers trained to predict O-linked glycosylation sites for which no obvious local sequence motifs exist, at a false positive rate of 0.15, the ensemble of SVMs has 6% greater sensitivity than the single SVM (the true positive rate of the single SVM is 0.78 whereas that of the ensemble of SVMs is 0.84) (Figure 2). For *θ *= 0.5, the estimated numbers TP, FN, FP, and TN of the single SVM are 1160, 937, 560, 10320 respectively, whereas those of the ensemble of SVMs are 1421, 676, 811, 10069. Thus, the single SVM achieves 0.88 accuracy, 0.55 Matthews correlation coefficient, 0.55 sensitivity, 0.67 specificity, 0.61 F-Measure, and 0.88 AUC, and the ensemble of SVMs achieves 0.89 accuracy, 0.59 Matthews correlation coefficient, 0.68 sensitivity, 0.64 specificity, 0.66 F-Measure, and 0.91 AUC (Table 2).

**Table 2:** Performance of classifiers trained to predict O-linked glycosylation sites

Performance Measure	SingleSVM	EnsembleSVM	BalancedSVM
Accuracy	0.88	**0.89**	0.85
MCC	0.55	**0.59**	0.57
Sensitivity	0.55	0.68	**0.80**
Specificity	**0.67**	0.64	0.53
F-Measure	0.61	**0.66**	0.64
AUC	0.88	**0.91**	0.90

Results obtained for O-linked glycosylation using single SVM from unbalanced data (singleSVM), ensemble of SVMs (EnsembleSVM), and single SVM from balanced data (BalancedSVM) for the classification threshold *θ *= 0.5 on the output probability of the classifier. The classifiers are trained on information derived from the target amino acid residue and its sequence neighbors.

In the case of classifiers trained to predict C-linked glycosylation sites (Figure 3) at a false positive rate of 0.2, the ensemble of SVMs has 17% greater sensitivity than the single SVM. For *θ *= 0.5, the estimated numbers TP, FN, FP, and TN of the single SVM are 35, 12, 9, 64 respectively, whereas those of the ensemble of SVMs are 37, 10, 11, 62. The single SVM achieves 0.83 accuracy, 0.63 Matthews correlation coefficient, 0.74 sensitivity, 0.80 specificity, 0.77 F-Measure, and 0.88 AUC, and the ensemble of SVMs achieves 0.83 accuracy, 0.63 Matthews correlation coefficient, 0.79 sensitivity, 0.77 specificity, 0.78 F-Measure, and 0.91 AUC (Table 3).

**Table 3:** Performance of classifiers trained to predict C-linked glycosylation sites

Performance Measure	SingleSVM	EnsembleSVM	BalancedSVM
Accuracy	**0.83**	**0.83**	**0.83**
MCC	**0.63**	**0.63**	**0.63**
Sensitivity	0.74	**0.79**	0.77
Specificity	**0.80**	0.77	0.78
F-Measure	0.77	**0.78**	0.77
AUC	0.88	**0.91**	0.89

Results obtained for C-linked glycosylation using single SVM from unbalanced data (singleSVM), ensemble of SVMs (EnsembleSVM), and single SVM from balanced data (BalancedSVM) for the classification threshold *θ *= 0.5 on the output probability of the classifier. The classifiers are trained on information derived from the target amino acid residue and its sequence neighbors.

### An ensemble of Support Vector Machines outperforms a single Support Vector Machine trained on balanced data on the glycosylation site prediction task

For each glycosylation type considered in this study, N-, O-, and C-linked glycosylation, we also compared the performance of the ensemble of SVM classifiers with that of a single SVM classifier trained on a balanced training set (obtained by sampling a number of non-glycosylation sites equal to the number of glycosylation sites) and evaluated on a test set (where the distribution of glycosylation and non-glycosylation sites corresponds to the original distribution). Note that it is important to evaluate the classifier on a dataset that reflects the distribution of glycosylation and non-glycosylation sites in the original dataset and *not *a dataset with an altered distribution.

**Figure 4:**
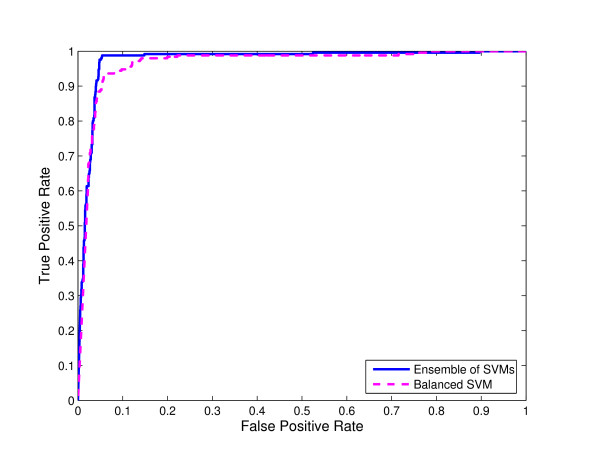
**Comparison of ensemble of SVMs and single SVM from balanced data for N-linked glycosylation using local sequence identity**. ROC curves for ensemble of SVMs and single SVM trained on the "altered" distribution of the data obtained by randomly selecting a subset of non-glycosylation sites equal in size with the set of glycosylation sites for N-linked glycosylation using local sequence identity with 0/1 String Kernel.

**Figure 5:**
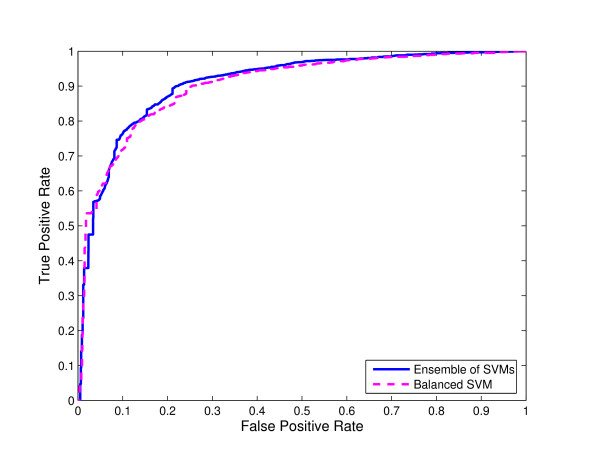
**Comparison of ensemble of SVMs and single SVM from balanced data for O-linked glycosylation using local sequence identity**. ROC curves for ensemble of SVMs and single SVM trained on the "altered" distribution of the data obtained by randomly selecting a subset of non-glycosylation sites equal in size with the set of glycosylation sites for O-linked glycosylation using local sequence identity with 0/1 String Kernel.

**Figure 6:**
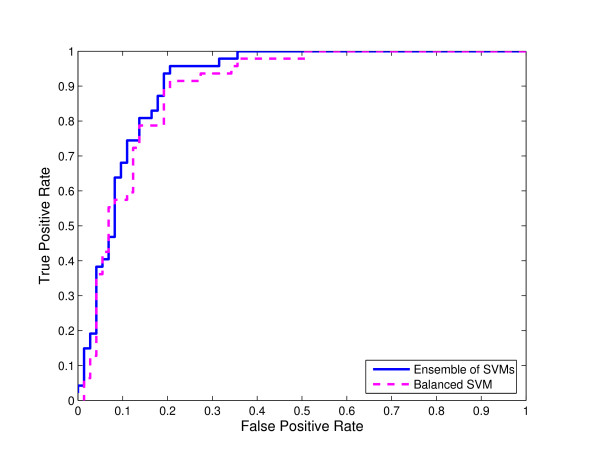
**Comparison of ensemble of SVMs and single SVM from balanced data for C-linked glycosylation using local sequence identity**. ROC curves for ensemble of SVMs and single SVM trained on the "altered" distribution of the data obtained by randomly selecting a subset of non-glycosylation sites equal in size with the set of glycosylation sites for C-linked glycosylation using local sequence identity with 0/1 String Kernel.

We compared the ROC curves for both ensemble of SVMs and single SVM trained on balanced data using local sequence information (the amino acid identity) with 0/1 String Kernel, for N-, O-, and C-linked glycosylation prediction tasks (Note that the ensemble of SVMs is trained on the original distribution of the glycosylation data). The ROC curves of ensembles of SVM classifiers for N-linked, O-linked, and C-linked glycosylation sites *dominate *the ROC curves for their single SVM counterparts for reasonably high values of sensitivity (Figures 4, 5, and 6 respectively).

For N-, O-, and C-linked glycosylation prediction tasks, the AUC of the ensemble of SVMs is larger than that of the corresponding single SVM (Tables 1, 2, and 3 respectively).

The results of this experiment show that simply balancing the training data used to train a single SVM classifier does not yield a classifier that performs as well as an ensemble of SVM classifiers. For example, in the case of single SVM trained on balanced data to predict O-linked glycosylation sites, the measured TP, FN, FP, and TN for the threshold *θ *= 0.5 are 1668, 429, 1477, and 9403 respectively. Thus, a single SVM trained on balanced data correctly identifies a larger fraction of glycosylation sites than the ensemble of SVMs, but does so at the cost of falsely predicting a greater fraction of non-glycosylation sites as glycosylation sites (the rate of false positive predictions for single SVM trained on balanced data is 0.14 as compared to 0.07 for an ensemble of SVMs).

## Discussion

### Performance of ensembles of Support Vector Machines on the task of predicting glycosylation sites

In this study, we explored ensembles of SVM classifiers trained on the "natural" distribution of the data extracted from the original glycoprotein sequence dataset to accurately discriminate between glycosylation and non-glycosylation sites in a protein sequence, for N-, O-, and C-linked glycosylation prediction tasks, using local sequence information (the amino acid identity) with 0/1 String Kernel.

An ensemble of SVMs is a collection of SVM classifiers, each trained on a *balanced *subsample of the training data. The prediction of the ensemble is computed from the predictions of the individual SVM classifiers. We performed sequence-based *k*-fold cross-validation [26,27] to estimate the *generalization accuracy *of the predictive models (i.e. the accuracy of the predictive models on the test set).

We found that ensembles of SVMs outperform both single SVM trained on unbalanced data and single SVM trained on balanced data.

In Figures 1, 2 and 3, we compared the Receiver Operating Characteristic (ROC) curves for ensemble of SVMs and single SVM trained on unbalanced data for N-, O-, and C-linked glycosylation prediction tasks respectively. The single SVM as well as the ensemble of SVMs are trained on the "natural" distribution of the data extracted from the original glycoprotein sequence dataset. As illustrated in the figures, the ROC curves of the ensembles of SVMs *dominate *the ROC curves of their single SVM counterparts.

In Figures 4, 5 and 6, we compared the ROC curves for ensemble of SVMs and single SVM trained on balanced data for N-, O-, and C-linked glycosylation prediction tasks respectively. The single SVM is trained on the "altered" distribution of the data obtained by randomly selecting a subset of non-glycosylation sites equal in size with the set of glycosylation sites, whereas the ensemble of SVMs is trained on the "natural" distribution of the glycosylation data. Again, the ROC curves of the ensembles of SVMs *dominate *the ROC curves of their single SVM counterparts for reasonably high values of sensitivity.

We also explored ensembles of SVMs using local sequence identity with Substitution Matrix String Kernel [28-30] for N-, O-, and C-linked glycosylation prediction tasks. We found that ensembles of SVMs using local sequence identity with Substitution Matrix String Kernel do not yield improvement over ensembles of SVMs using local sequence identity with 0/1 String Kernel.

We compared the performance of SVM (single and ensemble) classifiers using evolutionary information with Polynomial Kernel [31]. The feature vector representation was computed based on multiple sequence alignment profiles produced by *PSI-BLAST*, a tool that searches a large sequence database for sequence similarities [32]. The ROC curves for ensemble of SVMs *dominate *the ROC curves of single SVM for N-, O-, and C-linked glycosylation. Interestingly, ensembles of SVM classifiers trained using evolutionary information do not perform better than those trained using local sequence identity (Additional file 1).

### Performance of ensembles of Naive Bayes classifiers on the task of predicting glycosylation sites

In addition to ensembles of SVMs and single SVM classifiers, we trained ensembles of Naive Bayes and single Naive Bayes classifiers [33] on the original distribution of the data to identify putative glycosylation sites in a glycoprotein sequence. Naive Bayes classifiers offer a computationally efficient approach to training classifiers that are easier to understand than SVM or ensembles of SVMs for a variety of classification problems. We found that the performance of single Naive Bayes is similar to that of the ensemble of Naive Bayes classifiers as well as to that of the single SVM trained on unbalanced data (Additional file 2).

### Performance of ensembles of Support Vector Machines on the task of predicting glycosylation sites for a user-specified classification threshold

The ROC curves show the tradeoff between the rate of *true positive *predictions and the rate of false positive predictions for any user-specified classification threshold *θ *∈ [0, 1]. Hence, the estimated numbers of true positives, false negatives, false positives, and true negatives depend on how this classification threshold *θ *on the ROC curve is chosen. The threshold *θ *can be chosen to optimize a given performance measure (e.g. F-Measure, Matthews correlation coefficient) on the training data (see Methods section for further details). When *θ *was chosen to optimize the F-Measure, the results obtained with it are moderately better than the results obtained with the default value of *θ *= 0.5.

### Ensembles of Support Vector Machine classifiers -an approach to dealing with the unbalanced and large glycoprotein dataset

The glycoprotein dataset is highly unbalanced, i.e., the number of negative instances (S, T, N or W sites that are not known to be glycosylation sites) is much larger compared to the number of positive instances (S, T, N or W sites experimentally validated to be glycosylation sites). Unbalanced datasets present a challenge for Support Vector Machine classifiers that are trained to optimize the generalization accuracy.  They generally perform rather poorly on the minority class. Hence, if accurate classification of instances from the minority class is important, a common approach is to change the distribution of positive and negative instances during training by randomly selecting a subset of the training data for the majority class [22]. However, this makes it difficult to reliably identify the majority of the glycosylation sites without falsely predicting non-glycosylation sites as glycosylation sites. In addition, this approach fails to utilize all of the information available in the training data extracted from the original glycoprotein sequence dataset.

Results presented here demonstrate that a better approach is to construct an ensemble of SVM classifiers [17,18], each classifier being trained on a *balanced *subsample of the training data. The SVM classifiers in the ensemble are thus trained on different subsets of the training data. A sample instance is misclassified by the ensemble if a majority of the SVM classifiers in the ensemble misclassify it. When the errors made by the individual classifiers are uncorrelated, the predictions of the ensemble of classifiers are often more reliable.

The glycoprotein dataset is also very large i.e., it contains a large number of instances (Table 4). Large datasets present a computational challenge for machine learning algorithms such as SVM which solves a dual quadratic optimization problem to find the decision function. The use of an ensemble of SVM classifiers, each trained on a small subset of the training data significantly reduces the overall training time of a single SVM classifier trained on the entire training data. Construction of ensembles of classifiers makes SVM applicable to large datasets that would otherwise be considered "impractical" for training a single SVM classifier.

**Table 4:** Number of positive and negative sites used in our experiments for each of the three types of glycosylation considered

Glycosylation Type	Number of Positive Sites	Number of Negative Sites	Total Number of Sites
N-linked(N)	251	1430	1681
O-linked(S/T)	2097	10880	12977
C-linked(W)	47	73	120
Total	2395	12383	14778

The exact number of positive and negative instances for each of the three types of glycosylation considered for a window size of 21 (e.g., the actual number of positive and negative N sites for N-linked glycosylation, S/T sites for O-linked glycosylation, and W sites for C-linked glycosylation used in experiments).

### Comparison with previous work

In comparing the ensemble of SVM classifiers with the previous work on the glycosylation prediction task, we focused on the SVM approach presented in Li et al. [22]. The authors in [22] developed a system using SVM classifiers in order to predict O-linked glycosylation sites. There are four key differences between their approach and ours: in the datasets used, in the selection of *negative *examples, in the evaluation procedures, and in the methods used. We describe the differences in more detail in what follows.

First, the glycoprotein dataset used in [22] is extracted from SWISS-PROT/UniProt6.1 [34] and contains only mammalian glycoprotein sequences that have "mucin-type" O-linked glycosylation annotations. We use a glycoprotein dataset extracted from O-GlycBase v6.00 [35], a resource containing experimentally verified glycosylation sites compiled from protein databases and literature. Our dataset contains glycoprotein sequences from diverse eukaryotic organisms, (e.g., mammalian, insect, fungal), with three types of glycosylation annotations: N-linked, O-linked, and C-linked glycosylation annotations with a large variety of glycans (not just mucin-type).

A second difference between our approach and that of [22] has to do with the selection of *negative *examples (non-glycosylation sites) in the dataset. The negative examples in the dataset of [22] correspond to S/T sites sampled from mammalian protein sequences that lack annotation of glycosylation sites. In contrast, the negative examples in our dataset correspond to S/T sites for which no experimental evidence of glycosylation exists and are extracted from protein sequences that contain at least one experimentally verified glycosylation site. The underlying rationale for this choice is that the resulting negative examples are more likely to be non-glycosylation sites than the randomly extracted S/T sites from protein sequences with no experimentally verified glycosylation sites: total absence of experimentally verified glycosylation sites could simply mean that the sequence may not have been experimentally analyzed.

A third difference between our approach and that of Li et al. [22] has to do with the procedure used for performance evaluation. The positive and negative instances in the dataset used in [22] (sequence windows of length 41 with the target residue in the middle and 20 neighboring residues on each side) are filtered such that no two windows share sequence identity greater than 40%. "Leave-one-out" *window-based *cross-validation is performed to evaluate their classifiers. The instances in our dataset are sequence windows of length 21 with the target residue in the middle and 10 neighboring residues on each side. We have used instead, *sequence-based *5-fold cross-validation to evaluate our classifiers. As noted in [36], window-based cross-validation is likely to yield overly optimistic estimates of commonly used performance measures, such as Accuracy and Matthews Correlation Coefficient, relative to the estimates obtained using sequence-based cross-validation. Because classifiers trained on labeled sequence data have to predict the labels for residues in a novel glycoprotein sequence, the estimates obtained using sequence-based cross-validation provide more realistic estimates of the performance of a classifier than those obtained using window-based cross-validation.

A fourth key difference between the approach of Li et al. [22] and our approach has to do with the machine learning methods used. Li et al. used a *single SVM*. To get around the bias of SVM towards the negative class due to highly unbalanced dataset (larger number of negative instances relative to the number of positive instances), they experimented with different ratios of positive and negative instances to train SVM classifiers. That is, the number of negative instances is 1, 2, 3, 4, or 5 times the number of positive instances. Instead, we used an *ensemble of SVM classifiers*, trained on the original distribution of the data extracted from the original glycoprotein sequence dataset, with each SVM in the ensemble trained on a *balanced *subsample of the training data.

Because of the differences between our study and the study of Li et al. [22] noted above, it is not especially meaningful to directly compare the results of their study with ours. However, in the case of O-linked glycosylation sites, because the *SVM based on 0/1 system *in [22] is the same as the *single SVM with 0/1 String Kernel from balanced data *in our study, it is worth noting that the ensemble of SVMs outperforms single SVM in predicting O-linked glycosylation sites. The ROC curve of the ensemble of SVMs *dominates *the ROC curve of single SVM for reasonably high values of sensitivity (Figure 5). Moreover, the ensemble of SVMs achieves a larger AUC than the single SVM, and thus a larger overall probability of correct prediction for O-linked glycosylation sites (Table 2).

## Conclusion

Glycosylation plays important roles in protein folding, protein localization, trafficking, cell-cell interaction, developmental processes, etc [1-4]. With the rapid increase in the amount of data (e.g., protein sequences) there is a growing need for reliable procedures to accurately identify glycosylation sites.

In this study, we have presented a successful application of machine learning methods to identification of glycosylation sites from amino acid sequence of proteins. Specifically, we systematically evaluated single Support Vector Machines, as well as ensembles of Support Vector Machines in a sequence-based *k*-fold cross-validation setup [26,27,36]. The results of our experiments demonstrate that ensembles of SVMs outperform single SVMs in terms of a range of standard measures for comparing the performance of classifiers. The reliability with which N-, O-, and C-linked glycosylation sites are predicted in this study suggests that these classifiers, available online [37], can provide valuable information to guide experimental investigations. As more data from high-throughput experimental glycomics projects become available, it should be possible to further improve the reliability of predictions. Such data are needed to develop models that not only predict the sites of glycosylation, but that also capture the spatial and temporal dynamics of protein glycosylation that regulate developmental and immunological processes of clinical importance.

## Methods

### O-GLYCBASE Dataset

The dataset used in our experiments comes from O-GlycBase, a resource containing experimentally verified glycosylation sites compiled from protein databases and literature. The dataset is available online [16]. O-GlycBase v6.00 [35] contains no identical protein sequences, unless there are conflicts in the glycosylation data. It has 242 glycoproteins from different species: human, mouse, bovine, rat, insect, worm, horse, etc. A protein was included in the dataset if it had at least one experimentally verified O-, or C-linked glycosylation site. An entry in the database gives information about the glycan involved, the species, experimentally verified N, S/T, W glycosylation sites, literature references, protein sequence, http-linked cross-references to other protein sequence databases (e.g., SWISS-PROT, PIR, etc).

### Dataset Construction

After processing the O-GlycBase dataset, 216 glycoprotein entries are left in our dataset (we did not include proteins without an existent http-linked cross-reference to SWISS-PROT).

Based on the types of glycosylation considered in this study, three datasets are constructed from the 216 glycoprotein sequences: N-linked, O-linked, and C-linked datasets, each containing protein sequences that have at least one experimentally verified N-linked, O-linked, and C-linked glycosylation sites, respectively. Thus, N-linked dataset contains 86 protein sequences, O-linked dataset contains 205 protein sequences, and C-linked dataset contains 11 protein sequences.

As mentioned before, glycosylation is a site-specific process. It occurs on one of the four residues N, S, T, and W. However, not all of these residues in a protein sequence are actually modified by glycosylation. Therefore, we represent N sites (in N-linked dataset), S, T sites (in O-linked dataset), and W sites (in C-linked dataset) experimentally verified to be glycosylation sites as positive instances and N sites (in N-linked dataset), S, T sites (in O-linked dataset), and W sites (in C-linked dataset) not shown experimentally to be either glycosylation or non-glycosylation sites as negative instances. The resulting datasets contain very many negative instances, some of them in fact false negatives, (they may be discovered to be glycosylation sites in the future). We extract negative instances from sequences that have at least one experimentally verified glycosylation site because only a small fraction of N, S, T, and W residues are glycosylated. The protein sequences with no experimentally validated glycosylation sites may not have been analysed yet.

Overall, there are 2483 glycosylation sites composed of 254 N sites, 2180 S/T sites, and 49 W sites and 12935 non-glycosylation sites composed of 1469 N sites, 11388 S/T sites, and 78 W sites.

In addition to being a site-specific process, glycosylation is also an enzymatic process. It has been observed [9,10] that the enzymes involved (the transferases) recognize a glycosylation site based on the residues surrounding the target. To exploit this observation, we use a local window with each glycosylation or non-glycosylation site in the middle and *n *sequence neighbors on each side to further represent positive and negative instances, respectively. We denote by *s *= *s*_-*n*_*s*_-*n*+1 _⋯ *s*_-1_*s*_0_*s*_1 _⋯ *s*_*n*-1_*s*_*n *_a local window of length 2*n *+ 1, with *s*_0 _∈ {*N*, *S*, *T*, *W*}, *s*_*i *_∈ Σ, for *i *= -*n*, ⋯, *n*, *i *≠ 0 and *s *∈ Σ*, where Σ represents the amino acid alphabet. We ignored the sites close to N- and C-terminals. Table 4 shows the exact number of positive and negative instances for each of the three types of glycosylation considered in this study for a window length of 21 (*n *= 10).

### Support Vector Machine Classifier

Support Vector Machine (SVM) classifier is one of the most effective machine learning algorithms for many complex binary classification problems [31]. SVM is a supervised learning algorithm that belongs to the class of discriminative models.

Given a set of labeled inputs (**x**_*i*_, *y*_*i*_)_*i *= 1,⋯,*l*_, **x**_*i *_∈ **R**^*d *^and *y*_*i *_∈ {-1, +1}, learning an SVM classifier is equivalent to learning a decision function *f*(**x**) whose sign represents the class assigned to input **x**. This can be achieved by solving a dual quadratic optimization problem.

In the case of the linear SVM algorithm, when the training data is separable, it is possible to find linear decision functions *f*(**x**) = <**x**, **w**> + *b*, **w **∈ **R**^*d *^and *b *∈ **R **that accurately discriminate between positive and negative labeled inputs. Among these functions, SVM selects the one that minimizes ||**w**||^2^/2, which is equivalent to optimizing **w **and *b *such that the "margin" of separation (the distance) between the two classes is maximized. During classification, an unlabeled input **x**_*test *_is classified based on the sign of the decision function, *sgn*(*f*(**x**_*test*_)) (e.g., if *f *(**x**_*test*_) > 0 then **x**_*test *_is assigned to the positive class; otherwise **x**_*test *_is assigned to the negative class) [38].

When the training data is non-separable, the linear SVM algorithm does not find a feasible solution. In this case, an extra cost for errors can be assigned by introducing a set of positive slack variables *ξ*_*i*_, *i *= 1,⋯, *l *in the constraints of the optimization problem. The slack variables *ξ*_*i *_measure the extent to which the constrains are violated. SVM selects the decision function that minimizes ||*w*||^2^/2 + *C*(∑_*i*_*ξ*_*i*_)^*k*^, where *C *is a user parameter. The larger the value of *C*, the higher the penalty assigned to errors.

In the case of the nonlinear SVM algorithm, a linear decision function *f*(**x**) in the *d*-dimensional input space cannot be learned. The SVM algorithm works by mapping the labeled inputs into a (possibly) higher-dimensional *feature space *through an appropriate feature map, **x**_*i *_→ Φ(**x**_*i*_), *i *= 1,⋯,*l*, where a linear decision function can be found. Rather than explicitly computing the feature vector for each input **x**_*i*_, the mapping is defined implicitly via a *kernel function K*(**x**_*i*_, **x**_*j*_) = < Φ(**x**_*i*_), Φ(**x**_*j*_) >, *i*, *j *= 1,⋯,*l *that satisfies the Mercer's Condition [31]. The *kernel function *is evaluated for each pair of inputs, and specifies a similarity measure between them.

In this study, the *kernel function *that we used with SVM classifiers is *0/1 String Kernel*. The input of the classifiers is local sequence identity (the target amino acid residue and its sequence neighbors).

In order to obtain probabilistic outputs from SVM, i.e. the probability that the unlabeled input **x**_*test *_belongs to a certain class, *P*(*y*_*i*_|**x**_*test*_), we built a logistic model to map the outputs of the SVM to estimated probabilities [39].

For our experiments, we used the SVM algorithm implementation available in Weka [40]. The user parameter *C *was set to 1.0 (the default value).

#### 0/1 String Kernel

Given two local windows *s *= *s*_-*n*_*s*_-*n*+1 _⋯ *s*_-1_*s*_0_*s*_1 _⋯ *s*_*n*-1_*s*_*n *_and *t *= *t*_-*n*+1 _⋯ *t*_-1_*t*_0_*t*_1 _⋯ *t*_*n*-1_*t*_*n*_, the 0/1 String Kernel specifies a similarity measure between them based on their identities. Formally, this kernel is defined as:

K(s,t)=(∑i=−nnI[si=ti])p
 MathType@MTEF@5@5@+=feaafiart1ev1aaatCvAUfKttLearuWrP9MDH5MBPbIqV92AaeXatLxBI9gBaebbnrfifHhDYfgasaacPC6xNi=xI8qiVKYPFjYdHaVhbbf9v8qqaqFr0xc9vqFj0dXdbba91qpepeI8k8fiI+fsY=rqGqVepae9pg0db9vqaiVgFr0xfr=xfr=xc9adbaqaaeGacaGaaiaabeqaaeqabiWaaaGcbaGaem4saSKaeiikaGIaem4CamNaeiilaWIaemiDaqNaeiykaKIaeyypa0ZaaeWaaeaadaaeWbqaaiabdMeajjabcUfaBjabdohaZnaaBaaaleaacqWGPbqAaeqaaOGaeyypa0JaemiDaq3aaSbaaSqaaiabdMgaPbqabaGccqGGDbqxaSqaaiabdMgaPjabg2da9iabgkHiTiabd6gaUbqaaiabd6gaUbqdcqGHris5aaGccaGLOaGaayzkaaWaaWbaaSqabeaacqWGWbaCaaaaaa@49DE@

where *I*[·] is the indicator function; that is, *I*[*s*_*i *_= *t*_*i*_] = 1 if the amino acids on the *i*^*th *^position of the two local windows are the same, *s*_*i *_= *t*_*i*_, and *I*[*s*_*i *_= *t*_*i*_] = 0, otherwise. The higher the value of the kernel *K*(*s, t*), the more similar the local windows *s *and *t *are.

An explicit feature vector representation Φ(*s*) of a local window *s *can be easily computed in the following way: each amino acid in the local window is mapped to a 20-position binary vector with 1 on the position corresponding to the current amino acid and 0 on all the other positions, assuming a certain order among the 20 possible amino acids. That is, for each *i *= -*n*, ⋯,*n *and *j *= 1, ⋯, 20, (Φ(*s*))_*ij *_= 1 if the amino acid *s*_*i *_in the local window *s *is the same as the *j*^*th *^amino acid in Σ and (Φ(*s*))_*ij *_= 0 otherwise. Note that ∑j=120(Φ(s))ij=1
 MathType@MTEF@5@5@+=feaafiart1ev1aaatCvAUfKttLearuWrP9MDH5MBPbIqV92AaeXatLxBI9gBaebbnrfifHhDYfgasaacPC6xNi=xH8viVGI8Gi=hEeeu0xXdbba9frFj0xb9qqpG0dXdb9aspeI8k8fiI+fsY=rqGqVepae9pg0db9vqaiVgFr0xfr=xfr=xc9adbaqaaeGacaGaaiaabeqaaeqabiWaaaGcbaWaaabCaeaacqGGOaakcqqHMoGrcqGGOaakcqWGZbWCcqGGPaqkcqGGPaqkdaWgaaWcbaGaemyAaKMaemOAaOgabeaaaeaacqWGQbGAcqGH9aqpcqaIXaqmaeaacqaIYaGmcqaIWaama0GaeyyeIuoakiabg2da9iabigdaXaaa@3E6E@, for each *i *= -*n*, ⋯, *n*. The explicit feature vector representation has been widely used in [22,41].

However, the implicit kernel definition and the explicit feature vector representation with the Polynomial Kernel are equivalent. They represent the number of times the residues in the same position of two local windows are identical [42].

In our experiments, we used *p *= 2 for the degree of the kernel function.

### Ensemble of SVM classifiers

An *ensemble of SVM classifiers *[17,18] is a collection of SVM classifiers, each trained on a *balanced *subsample of the training data (approximately equal number of positive and negative instances obtained by sampling with replacement from the entire training data). Note that the ensemble of SVM classifiers is trained and evaluated on the original distribution of the glycosylation data. The prediction of the ensemble of SVMs is computed from the predictions of the individual SVM classifiers. That is, during classification, for a new unlabeled input **x**_*test*_, each individual SVM classifier in the collection returns a probability *P*_*j*_(*y*_*i*_|**x**_*test*_), that **x**_*test *_belongs to a particular class *y*_*i*_, where *j *= 1,⋯, *m*, and *m *is the number of SVM classifiers in the collection. The ensemble estimated probability, *P*_*Ens*_(*y*_*i*_|**x**_*test*_) is obtained by:

PEns(yi|xtest)=1m∑jmPj(yi|xtest)
 MathType@MTEF@5@5@+=feaafiart1ev1aaatCvAUfKttLearuWrP9MDH5MBPbIqV92AaeXatLxBI9gBaebbnrfifHhDYfgasaacPC6xNi=xI8qiVKYPFjYdHaVhbbf9v8qqaqFr0xc9vqFj0dXdbba91qpepeI8k8fiI+fsY=rqGqVepae9pg0db9vqaiVgFr0xfr=xfr=xc9adbaqaaeGacaGaaiaabeqaaeqabiWaaaGcbaGaemiuaa1aaSbaaSqaaiabdweafjabd6gaUjabdohaZbqabaGccqGGOaakcqWG5bqEdaWgaaWcbaGaemyAaKgabeaakiabcYha8Hqabiab=Hha4naaBaaaleaacqWG0baDcqWGLbqzcqWGZbWCcqWG0baDaeqaaOGaeiykaKIaeyypa0tcfa4aaSaaaeaacqaIXaqmaeaacqWGTbqBaaWaaabCaeaacqWGqbaudaWgaaqaaiabdQgaQbqabaGaeiikaGIaemyEaK3aaSbaaeaacqWGPbqAaeqaaiabcYha8jab=Hha4naaBaaabaGaemiDaqNaemyzauMaem4CamNaemiDaqhabeaacqGGPaqkaeaacqWGQbGAaeaacqWGTbqBaiabggHiLdaaaa@57E9@

**Figure 7:**
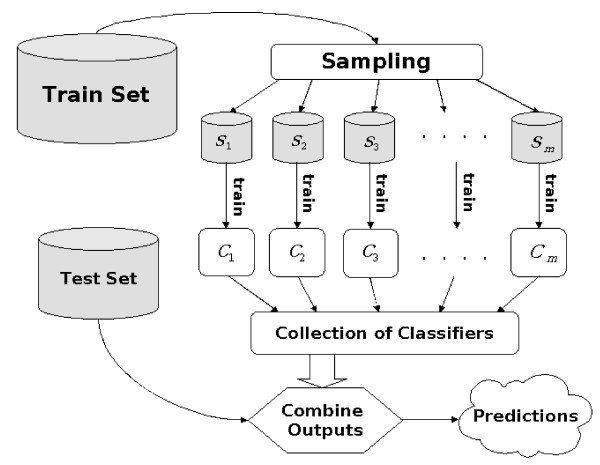
**Architecture of the ensemble of Support Vector Machine classifiers**. A collection of m SVM classifiers, each trained on a *balanced *subsample of the training data (approximately equal number of positive and negative instances obtained by sampling with replacement from the entire training data). The ensemble of SVM classifiers is trained and evaluated on the original distribution of the glycosylation data. The prediction of the ensemble of SVMs is computed from the predictions of the individual SVM classifiers.

In our experiments, we used *m *= 40. Each individual SVM classifier in the collection was trained on approximately l10
 MathType@MTEF@5@5@+=feaafiart1ev1aaatCvAUfKttLearuWrP9MDH5MBPbIqV92AaeXatLxBI9gBaebbnrfifHhDYfgasaacPC6xNi=xH8viVGI8Gi=hEeeu0xXdbba9frFj0xb9qqpG0dXdb9aspeI8k8fiI+fsY=rqGqVepae9pg0db9vqaiVgFr0xfr=xfr=xc9adbaqaaeGacaGaaiaabeqaaeqabiWaaaGcbaqcfa4aaSaaaeaacqWGSbaBaeaacqaIXaqmcqaIWaamaaaaaa@2FB0@ instances, where *l *represents the total number of training instances available to the ensemble. Figure 7 shows the architecture of the ensemble of SVM classifiers.

### Performance Evaluation

To assess the performance of our classifiers we report the following measures described in [24]: Accuracy, Matthews Correlation Coefficient (MCC), Sensitivity, and Specificity (also known as Recall and Precision), True Positive Rate (TPR) and False Positive Rate (FPR). If we denote true positives, false negatives, false positives, and true negatives by *TP*, *FN*, *FP*, and *TN *respectively, then these measures can be defined as follows:

Accuracy=TP+TNTP+FN+FP+TNMCC=TP⋅TN−FP⋅FN(TP+FN)(TP+FP)(TN+FP)(TN+FN)Sensitivity=TPTP+FN,Specificity=TPTP+FPTPR=TPTP+FN,FPR=FPFP+TN
 MathType@MTEF@5@5@+=feaafiart1ev1aaatCvAUfKttLearuWrP9MDH5MBPbIqV92AaeXatLxBI9gBaebbnrfifHhDYfgasaacPC6xNi=xI8qiVKYPFjYdHaVhbbf9v8qqaqFr0xc9vqFj0dXdbba91qpepeI8k8fiI+fsY=rqGqVepae9pg0db9vqaiVgFr0xfr=xfr=xc9adbaqaaeGacaGaaiaabeqaaeqabiWaaaGcbaqbaeqabqqaaaaabaGaeeyqaeKaee4yamMaee4yamMaeeyDauNaeeOCaiNaeeyyaeMaee4yamMaeeyEaKNaeyypa0tcfa4aaSaaaeaacqWGubavcqWGqbaucqGHRaWkcqWGubavcqWGobGtaeaacqWGubavcqWGqbaucqGHRaWkcqWGgbGrcqWGobGtcqGHRaWkcqWGgbGrcqWGqbaucqGHRaWkcqWGubavcqWGobGtaaaakeaacqqGnbqtcqqGdbWqcqqGdbWqcqGH9aqpjuaGdaWcaaqaaiabdsfaujabdcfaqjabgwSixlabdsfaujabd6eaojabgkHiTiabdAeagjabdcfaqjabgwSixlabdAeagjabd6eaobqaamaakaaabaGaeiikaGIaemivaqLaemiuaaLaey4kaSIaemOrayKaemOta4KaeiykaKIaeiikaGIaemivaqLaemiuaaLaey4kaSIaemOrayKaemiuaaLaeiykaKIaeiikaGIaemivaqLaemOta4Kaey4kaSIaemOrayKaemiuaaLaeiykaKIaeiikaGIaemivaqLaemOta4Kaey4kaSIaemOrayKaemOta4KaeiykaKcabeaaaaaakeaafaqabeqacaaabaGaee4uamLaeeyzauMaeeOBa4Maee4CamNaeeyAaKMaeeiDaqNaeeyAaKMaeeODayNaeeyAaKMaeeiDaqNaeeyEaKNaeyypa0tcfa4aaSaaaeaacqWGubavcqWGqbauaeaacqWGubavcqWGqbaucqGHRaWkcqWGgbGrcqWGobGtaaGaeiilaWcakeaacqqGtbWucqqGWbaCcqqGLbqzcqqGJbWycqqGPbqAcqqGMbGzcqqGPbqAcqqGJbWycqqGPbqAcqqG0baDcqqG5bqEcqGH9aqpjuaGdaWcaaqaaiabdsfaujabdcfaqbqaaiabdsfaujabdcfaqjabgUcaRiabdAeagjabdcfaqbaaaaaakeaafaqabeqacaaabaGaeeivaqLaeeiuaaLaeeOuaiLaeyypa0tcfa4aaSaaaeaacqWGubavcqWGqbauaeaacqWGubavcqWGqbaucqGHRaWkcqWGgbGrcqWGobGtaaGccqGGSaalaeaacqqGgbGrcqqGqbaucqqGsbGucqGH9aqpjuaGdaWcaaqaaiabdAeagjabdcfaqbqaaiabdAeagjabdcfaqjabgUcaRiabdsfaujabd6eaobaaaaaaaaaa@C68E@

Note that TPR is the same as Sensitivity.

In addition to these measures, we report the F-Measure [43], which is the harmonic mean of Precision and Recall.

F−Measure=2⋅Recall⋅PrecisionRecall+Precision
 MathType@MTEF@5@5@+=feaafiart1ev1aaatCvAUfKttLearuWrP9MDH5MBPbIqV92AaeXatLxBI9gBaebbnrfifHhDYfgasaacPC6xNi=xI8qiVKYPFjYdHaVhbbf9v8qqaqFr0xc9vqFj0dXdbba91qpepeI8k8fiI+fsY=rqGqVepae9pg0db9vqaiVgFr0xfr=xfr=xc9adbaqaaeGacaGaaiaabeqaaeqabiWaaaGcbaGaeeOrayKaeyOeI0Iaeeyta0KaeeyzauMaeeyyaeMaee4CamNaeeyDauNaeeOCaiNaeeyzauMaeyypa0tcfa4aaSaaaeaacqaIYaGmcqGHflY1cqqGsbGucqqGLbqzcqqGJbWycqqGHbqycqqGSbaBcqqGSbaBcqGHflY1cqqGqbaucqqGYbGCcqqGLbqzcqqGJbWycqqGPbqAcqqGZbWCcqqGPbqAcqqGVbWBcqqGUbGBaeaacqqGsbGucqqGLbqzcqqGJbWycqqGHbqycqqGSbaBcqqGSbaBcqGHRaWkcqqGqbaucqqGYbGCcqqGLbqzcqqGJbWycqqGPbqAcqqGZbWCcqqGPbqAcqqGVbWBcqqGUbGBaaaaaa@6750@

#### Receiver Operating Characteristic (ROC) Curve

For each classifier we draw the Receiver Operating Characteristic (ROC) curve, which plots the proportion of correctly classified positive instances, True Positive Rate (TPR), as a function of the proportion of incorrectly classified negative instances, False Positive Rate (FPR). Each point on the ROC curve represents a classification threshold *θ *∈ [0, 1] and corresponds to particular values of TPR and FPR. A site is predicted to be a glycosylation site if the output probability of a classifier, *P*(*y*_*i *_= +1|**x**_*test*_), is greater than *θ*, and a non-glycosylation site otherwise. The default value of *θ *is 0.5. Varying the threshold *θ *gives a tradeoff between TPR and FPR.

#### Area Under the ROC Curve (AUC)

To evaluate how good a classifier is to discriminate between the positive and negative instances, we also report the Area Under the ROC Curve (AUC) on the test set, which represents the probability of correct classification [24,25]. That is, an AUC of 0.5 indicates a random discrimination between positives and negatives (a random classifier), while an AUC of 1 indicates a perfect discrimination (a very good classifier).

#### Sequence-Based K-Fold Cross-Validation Procedure

The above performance measures are computed based on *sequence-based k-fold cross-validation *procedure [26,27,36]. *K*-fold cross-validation [33] is an evaluation scheme considered by many authors to be a good method of estimating the *generalization accuracy *of a predictive algorithm (i.e. the accuracy of the predictive model on the test set).

During *sequence-based k-fold cross-validation*, the original dataset of glycoprotein sequences is randomly partitioned into *k *disjoint subsets of approximately equal size. The cross-validation is performed *k *different times. During the *i*^*th *^run, *i *= 1,⋯,*k*, the *i*^*th *^subset (the holdout set) is used for testing and the remaining *k *- 1 subsets are used for training. Each glycoprotein sequence in the dataset is used exactly once in the test set and *k *- 1 times in the training set. The results from the *k *different runs are then averaged.

For the ensemble of SVMs and single SVM classifiers trained on unbalanced data, the distribution of both training and test sets corresponds to the original distribution of glycosylation data. For single SVM classifiers trained on balanced data, the distribution of the training set is altered by sampling a number of negative instances equal to the number of positive instances, whereas the distribution of the test set corresponds to the original distribution of glycosylation data. Note that it is important to evaluate the classifier on a dataset that reflects the distribution of glycosylation and non-glycosylation sites in the original dataset and *not *a dataset with an altered distribution.

#### Threshold Selection

The glycoprotein dataset is highly unbalanced, i.e. the number of negative instances is much larger compared to the number of positive instances. When the dataset is unbalanced, the measure of accuracy is not a good indicator of the performance of the classifier because the classifier will be biased towards the class with the larger number of instances (negative class in our case). In such a setting, even a classifier that always labels instances as negatives would give a reasonably good accuracy, while performing unacceptably poor on the minority class (positive class in our case).

To avoid this problem, we select the classification threshold *θ *on the training set as follows: the training set is randomly partitioned into *p *disjoint subsets of approximately equal size. Next, the cross-validation is performed *p *different times. During the *j*^*th *^run, for *j *= 1, ⋯ ,*p*, the *j*^*th *^subset is used for testing and the remaining *p *- 1 subsets are used for training. After all the predictions are made available, the point on the ROC curve that gives the best F-Measure value is chosen as the classification threshold *θ*. Note that during this procedure, the classifier uses only the training data. A new instance **x**_*test *_is predicted as positive if *P*(*y*_*i *_= +1|**x**_*test*_) > *θ*, and negative otherwise.

For our experiments, we used *k *= *p *= 5.

## Authors' contributions

CC and JS carried out the computations. CC prepared an initial draft of the manuscript. CC, JS, and VH revised the manuscript based on reviewers comments. JS carried out server implementation. AS, DD, and VH participated in experimental design, discussions, manuscript preparation and revisions. All authors read and approved the final manuscript.

## Supplementary Material

Additional file 1 **Comparison of single versus ensemble of Support Vector Machine classifiers using evolutionary information with Polynomial Kernel**. ROC curves for single and ensemble of Support Vector Machine classifiers for N-, O-, and C-linked glycosylation using evolutionary information with Polynomial Kernel and the description of evolutionary information feature representation. Click here for file

Additional file 2 **Comparison of single versus ensemble of Naive Bayes classifiers and single Naive Bayes versus single SVM using local sequence information**. ROC curves for single Naive Bayes and ensemble of Naive Bayes classifiers and ROC curves for single Naive Bayes and single SVM for N-, O-, and C-linked glycosylation using local sequence information. Click here for file
