# Supplementary material for: Glycosylation site prediction using ensembles of Support Vector Machine classifiers
Source: BMC Bioinformatics. 2007 Nov 9;8:438. doi: 10.1186/1471-2105-8-438 (PMC2220009; doi:10.1186/1471-2105-8-438)
Supplement: Additional file 1 — Comparison of single versus ensemble of Support Vector Machine classifiers using evolutionary information with Polynomial Kernel. ROC curves for single and ensemble of Support Vector Machine classifiers for N-, O-, and C-linked glycosylation using evolutionary information with Polynomial Kernel and the description of evolutionary information feature representation. [file 1471-2105-8-438-S1.pdf]

Comparison of single versus ensemble of Support Vector Machine classifiers using evolutionary information with Polynomial Kernel

For N-, O-, and C-linked glycosylation, we trained ensembles of Support Vector Machine (SVM) classifiers using evolutionary information to predict whether or not a site in a protein sequence is a glycosylation site. An ensemble of SVMs is simply a collection of SVM classifiers, each trained on a *balanced* subsample of the training data. The prediction of the ensemble is computed from the predictions of the individual SVM classifiers (see Methods section in the manuscript for further details).

We compared the ROC curves for both ensemble of SVMs and single SVM using evolutionary information with Polynomial Kernel [1] for N-, O-, and C-linked glycosylation prediction tasks. As in the case of SVM classifiers trained using amino acid identity, the ROC curves of the ensembles of SVMs using evolutionary information for N-linked, O-linked, and C-linked glycosylation sites *dominate* the ROC curves for their single SVM counterparts (Figures 1, 2, and 3 respectively). That is, for any choice of false positive rate, the ensemble of SVMs offers a higher *true positive rate* than the single SVM for the same task. However, the ensembles of SVM classifiers trained using evolutionary information with Polynomial Kernel do not perform better than those using local sequence identity with 0/1 String Kernel (Figure 4).

For N-, O-, and C-linked glycosylation prediction tasks, the Area Under the ROC Curve (AUC) [2] is larger for the ensemble of SVMs than for the corresponding single SVM.

The feature vector representation is computed explicitly for each target amino acid residue and its sequence neighbors based on local and global multiple sequence alignment profiles produced by *PSI-BLAST*, a tool that searches a large sequence database for sequence similarities [3]. In the case of local multiple sequence alignment profiles, each local sequence (positive or negative window) is locally aligned with protein sequences in the *nr* (non-redundant) database and the local multiple sequence alignment profile is returned. In the case of global multiple sequence alignment profiles, each glycoprotein sequence is globally aligned with protein sequences in the *nr* (non-redundant) database and the global multiple sequence alignment profile is returned.

The results obtained using local multiple sequence alignment profiles are better than those obtained using global multiple sequence alignment profiles for N- and O-linked glycosylation, and worse than those obtained using global multiple sequence alignment profiles for C-linked glycosylation. The ROC curves

shown here for both ensemble of SVMs and single SVM using evolutionary information with Polynomial Kernel are based on local multiple sequence alignment profiles for N-, O-, and C-linked glycosylation prediction tasks.

We explored single SVM and ensemble of SVMs using evolutionary information with the Radial Basis Function (RBF) Kernel [1]. In general, the results obtained with the RBF Kernel do not outperform the results obtained with the Polynomial Kernel for N- and O-linked glycosylation. However, the RBF Kernel performs better than the Polynomial Kernel for C-linked glycosylation.

In our experiments, the Polynomial Kernel parameter θ was set to 0 and the exponent p to 2. The RBF parameter σ was set to 0.01. The parameter values for *PSI-BLAST* are 0.001 for the e-value threshold to include a sequence in the next iteration and 2 for the number of iterations.

References

1. Burges CJC: **A Tutorial on Support Vector Machines for Pattern Recognition**. *Data Mining and Knowledge Discovery* 1998, **2**:121–167.
2. Gribskov M, Robinson N: **The Use of Receiver Operating Characteristic (ROC) Analysis to Evaluate Sequence Matching**. *Comput. Chem.* 1996, **20**:25–33.
3. Altschul SF, Madden TL, Schaffer AA, Zhang J, Zhang Z, Miller W, Lipman DJ: **Gapped BLAST and PSI-BLAST: a new generation of protein database search programs**. *Nucleic Acids Res.* 1997, **25**(17):3389–402.

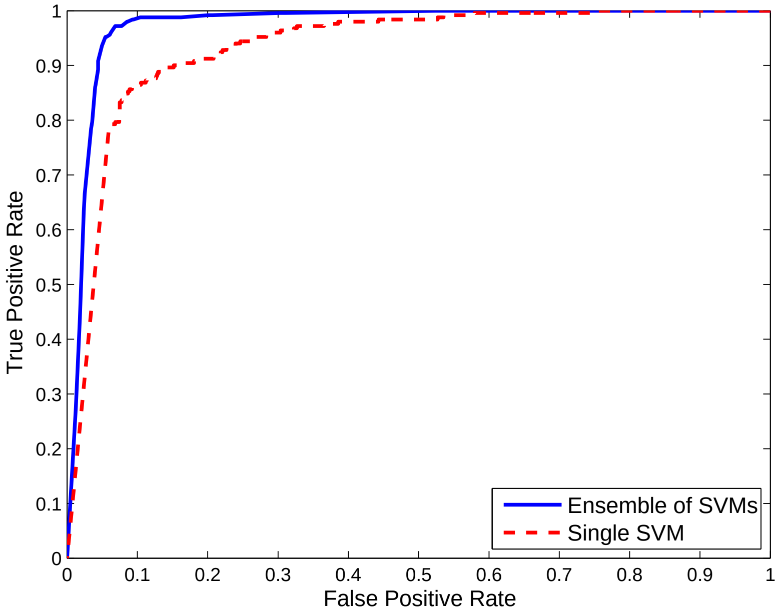

Figure 1: ROC curves for single and ensemble of Support Vector Machine classifiers for N-linked glycosylation using evolutionary information with Polynomial Kernel.

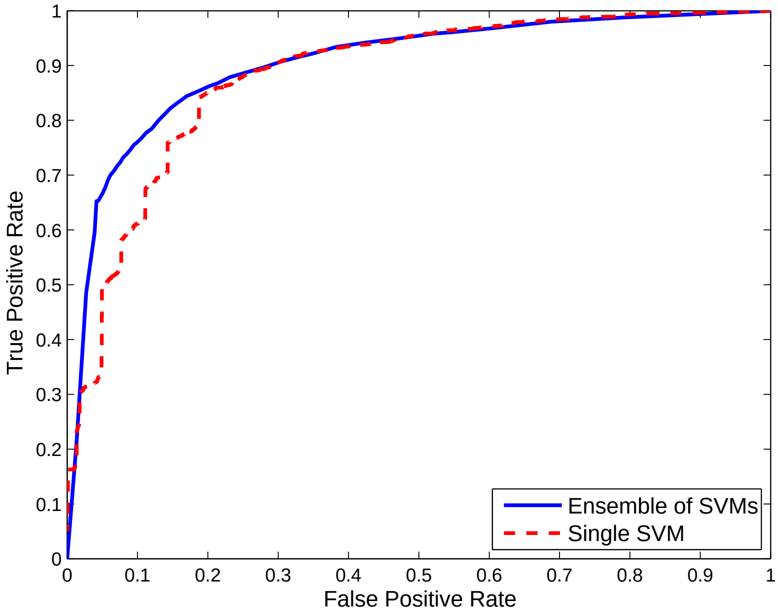

Figure 2: ROC curves for single and ensemble of Support Vector Machine classifiers for O-linked glycosylation using evolutionary information with Polynomial Kernel.

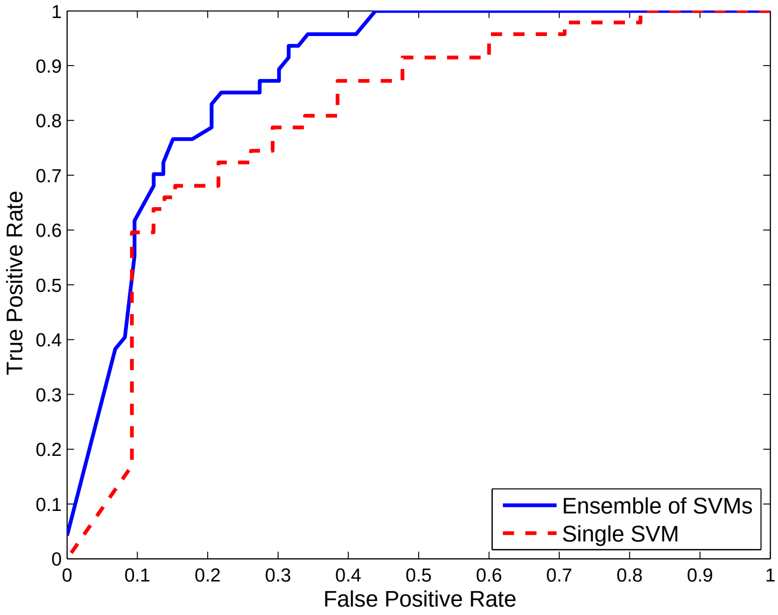

Figure 3: ROC curves for single and ensemble of Support Vector Machine classifiers for C-linked glycosylation using evolutionary information with Polynomial Kernel.

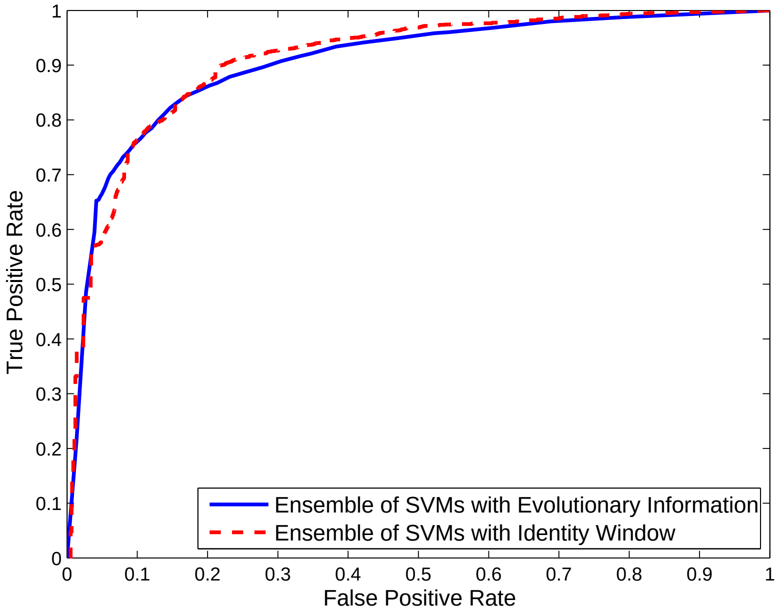

Figure 4: ROC curves for the ensemble of Support Vector Machine classifiers using amino acid identity window with 0/1 String Kernel and the ensemble of Support Vector Machine classifiers using evolutionary information with Polynomial Kernel in the case of O-linked glycosylation.
