# Supplementary material for: Glycosylation site prediction using ensembles of Support Vector Machine classifiers
Source: BMC Bioinformatics. 2007 Nov 9;8:438. doi: 10.1186/1471-2105-8-438 (PMC2220009; doi:10.1186/1471-2105-8-438)
Supplement: Additional file 2 — Comparison of single versus ensemble of Naive Bayes classifiers and single Naive Bayes versus single SVM using local sequence information. ROC curves for single Naive Bayes and ensemble of Naive Bayes classifiers and ROC curves for single Naive Bayes and single SVM for N-, O-, and C-linked glycosylation using local sequence information. [file 1471-2105-8-438-S2.pdf]

Performance of ensembles of Naive Bayes classifiers on the task of predicting glycosylation sites

For each glycosylation type considered in this study, N-, O-, and C-linked glycosylation, we trained ensembles of Naive Bayes and single Naive Bayes classifiers [1] on the original distribution of the data to identify putative glycosylation sites in a glycoprotein sequence. We compared the ROC curves for single Naive Bayes and ensemble of Naive Bayes using local sequence information (the amino acid identity) for N-, O-, and C-linked glycosylation prediction tasks. We found that the performance of single Naive Bayes is similar to that of the ensemble of Naive Bayes classifiers (Figures 1, 2, and 3 respectively).

Performance of Naive Bayes classifiers is similar to that of single Support Vector Machine classifiers

For N-, O-, and C-linked glycosylation, we trained Naive Bayes classifiers [1] to identify putative glycosylation sites in a glycoprotein sequence. Compared to Support Vector Machines, Naive Bayes classifiers are easier to understand and faster to train. Hence, when a better understanding of the output of the classifiers is desirable or faster training is required, Naive Bayes classifiers represent a better choice.

We compared the ROC curves for Naive Bayes and SVM using local sequence information (the amino acid identity), for N-, O-, and C-linked glycosylation prediction tasks (Figures 4, 5, and 6 respectively). Both Naive Bayes and SVM were trained on the "natural" distribution of the data extracted from the original glycoprotein sequence dataset. The figures illustrate that the performance of Naive Bayes is similar to that of SVM for all three tasks.

Performance of Naive Bayes classifiers on the task of predicting glycosylation sites using physicochemical properties of amino acids

Previous studies have shown that the use of physicochemical properties of amino acids in addition to amino acid identity can often improve the performance of classifiers [2]. Hence, we explored whether the use of physicochemical features, namely, the size of the molecule ("large", "small", or "tiny"), hydrophobicity ("yes", "no", or "partial"), polarity ("yes" or "no"), charge ("positive", "negative", or "none"), aliphaticity ("aliphatic" or "not aliphatic"), and aromaticity ("aromatic" or "not aromatic") [3] would improve the performance of the classifiers on the glycosylation site prediction task. Comparison of Naive Bayes classifiers using the identity of amino acids that are sequence neighbors of the target residue alone with Naive Bayes classifiers using the physicochemical properties of amino acid residues in addition to local

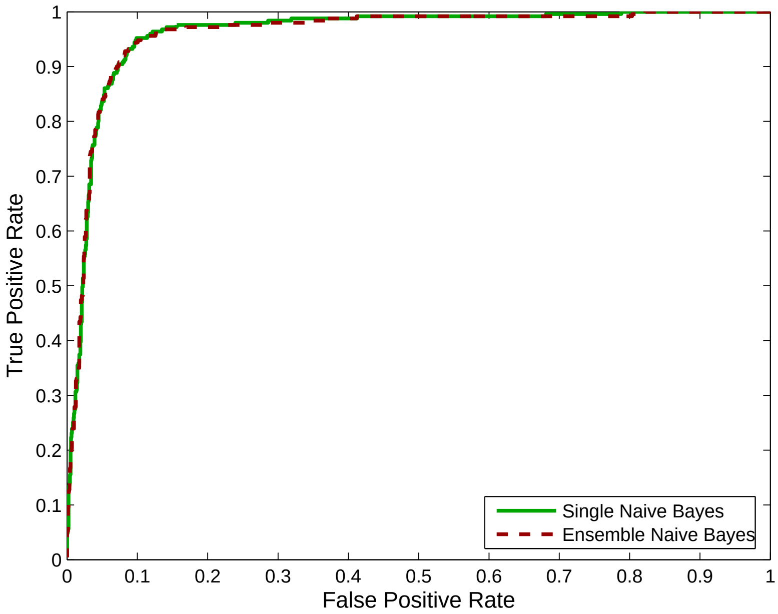

Figure 1: ROC curves for single and ensemble of Naive Bayes classifiers for N-linked glycosylation trained using local sequence identity.

sequence identity yields moderate improvement in the performance of the resulting classifiers.

For our experiments, we used Naive Bayes implementation available in Weka [4].

References

1. Mitchell TM: *Machine Learning.* McGraw Hill 1997.
2. Li X, Pan X: **New method for accurate prediction of solvent accessibility from protein sequence**. *Proteins: Structure, Function, and Genetics* 2001, **42**:1–5.
3. Betts M, Russell R: *Amino acid properties and consequences of subsitutions.* In Bioinformatics for Geneticists, Wiley 2003.
4. **Weka 3: Data Mining Software in Java** [http://www.cs.waikato.ac.nz/ml/weka/].

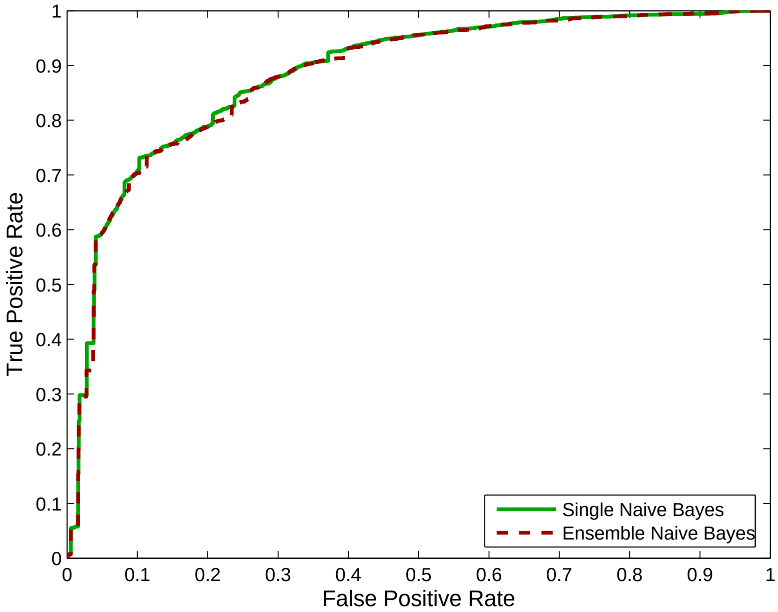

Figure 2: ROC curves for single and ensemble of Naive Bayes classifiers for O-linked glycosylation trained using local sequence identity.

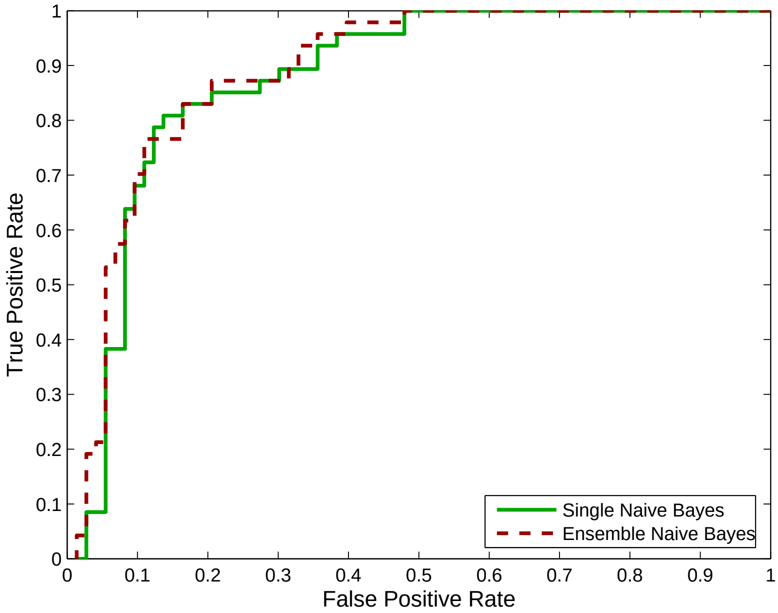

Figure 3: ROC curves for single and ensemble of Naive Bayes classifiers for C-linked glycosylation trained using local sequence identity.

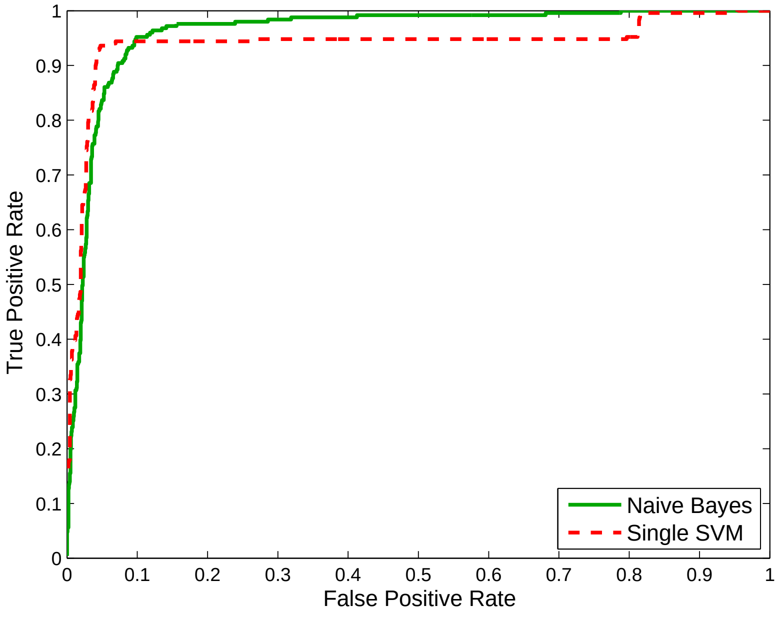

Figure 4: ROC curves for Naive Bayes and Support Vector Machine classifiers for N-linked glycosylation trained using local sequence identity.

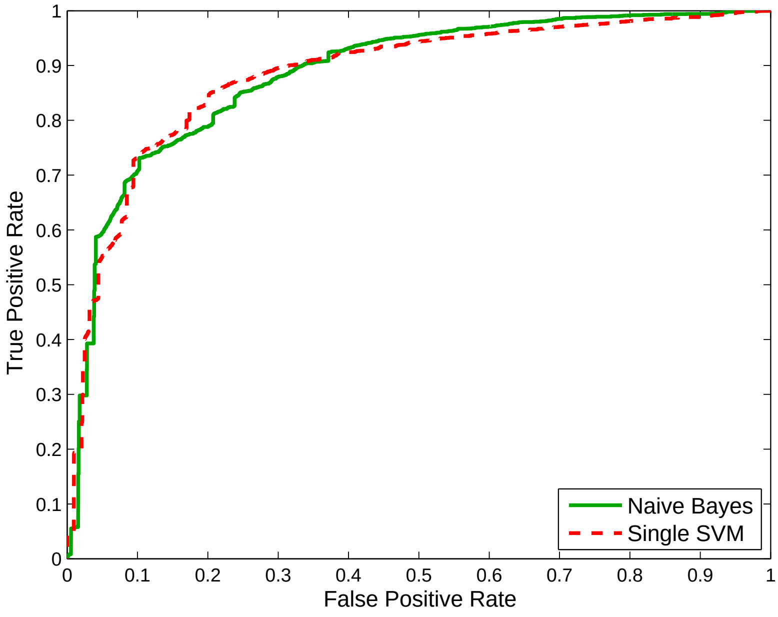

Figure 5: ROC curves for Naive Bayes and Support Vector Machine classifiers for O-linked glycosylation trained using local sequence identity.

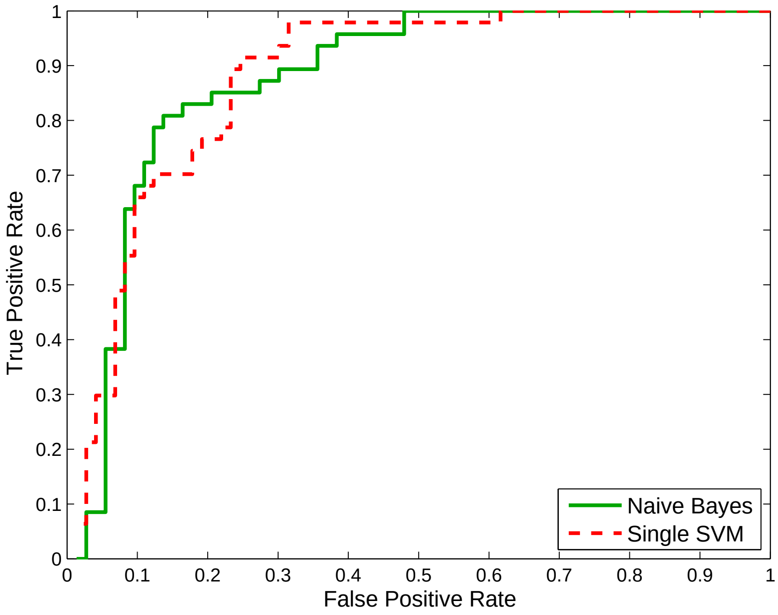

Figure 6: ROC curves for Naive Bayes and Support Vector Machine classifiers for C-linked glycosylation trained using local sequence identity.
